# Refractory Uterine Atony After Sequential Neuraxial Opioid Administration—A Case Report

**DOI:** 10.3390/reports9030227

**Published:** 2026-07-15

**Authors:** Ramona Celia Moisa, Nicoleta Negrut, Cezar Cristian Mihai Moisa, Denisa Florina But, Harrie Toms John, Paula Marian

**Affiliations:** 1 Clinic of Anaesthesia and Intensive Care, Pelican Clinic, Medicover Hospital, 4104869 Oradea, Romania; moisa.ramona.celia@didactic.uoradea.ro; 2 Doctoral School of Biomedical Sciences, Faculty of Medicine and Pharmacy, University of Oradea, 410087 Oradea, Romania; moisa.cezarcristianmihai@student.uoradea.ro (C.C.M.M.); harrie.tomsjohn@student.uoradea.ro (H.T.J.); 3 Department of Surgery, Faculty of Medicine and Pharmacy, University of Oradea, 410087 Oradea, Romania; 4 Department of Psycho-Neuroscience and Recovery, Faculty of Medicine and Pharmacy, University of Oradea, 410073 Oradea, Romania; 5 Department of Anesthesia and Intensive Care, Emergency Clinical Hospital of Târgu Mureș, 540136 Târgu Mureș, Romania; but.florina-denisa@stud17.umftgm.ro; 6 George Emil Palade University of Medicine, Pharmacy, Science, and Technology of Târgu Mureș, 540136 Târgu Mureș, Romania; 7 Department of Medical Disciplines, Faculty of Medicine and Pharmacy, University of Oradea, 410073 Oradea, Romania; paulamarian@uoradea.ro

**Keywords:** uterine atony, postpartum hemorrhage, neuraxial opioids, cesarean delivery, diagnostic challenge, case report

## Abstract

**Background and Clinical Significance:** Uterine atony is the most frequent cause of postpartum hemorrhage and remains a major contributor to maternal morbidity worldwide. Neuraxial opioids are routinely used as adjuvants for labor analgesia and cesarean delivery anesthesia; however, their possible influence on myometrial contractility remains incompletely clarified. We describe a severe case of refractory uterine atony after emergency cesarean delivery in a patient exposed sequentially to epidural fentanyl during labor and intrathecal morphine for cesarean anesthesia; **Case Presentation:** A 34-year-old primigravida at 39 weeks and 4 days of gestation presented with early labor that had begun less than one hour earlier. Epidural labor analgesia was provided with ropivacaine, and the total epidural fentanyl exposure was 100 mcg over an approximately 7 h catheter period. Labor was complicated by dysfunctional uterine activity and cervical dystocia despite 3 h and 30 min of oxytocin augmentation. Emergency cesarean delivery was performed under spinal anesthesia with hyperbaric bupivacaine and intrathecal morphine. After delivery of a healthy neonate and uncomplicated placental separation, the patient developed severe uterine atony with postpartum hemorrhage. Hemorrhage persisted despite uterotonic therapy, continuous uterine massage, hemostatic suturing, and B-Lynch compression suture. Blood loss, measured using the suction canister and estimated from surgical swabs, was approximately 3800 mL. Progressive hemodynamic instability required transfusion therapy, conversion to general anesthesia, norepinephrine support, and emergency total abdominal hysterectomy. The postoperative course was favorable, and the patient was discharged on the eighth postoperative day; **Conclusions**: This case illustrates the rapid progression and therapeutic complexity of refractory uterine atony after emergency cesarean delivery in the setting of dysfunctional labor, oxytocin augmentation, cesarean delivery, sequential neuraxial opioid exposure, and subsequent hemorrhagic instability. A possible contribution of sequential neuraxial opioid administration to impaired myometrial contractility cannot be excluded; however, causality cannot be established from a single case. Early recognition, structured escalation, transfusion support, and timely multidisciplinary surgical management remain essential in severe postpartum hemorrhage.

## 1. Introduction and Clinical Significance

Spinal anesthesia is currently considered the preferred anesthetic technique for most cesarean deliveries because of its rapid onset, reliable sensory block, and favorable maternal and neonatal safety profile [1]. Neuraxial opioids such as fentanyl and morphine are widely used as adjuvants to improve intraoperative and postoperative analgesia while reducing systemic opioid requirements [2,3,4]. Epidural fentanyl, a highly lipophilic opioid, provides rapid analgesia and is commonly combined with local anesthetics during labor [5]. Intrathecal morphine, because of its hydrophilic properties, offers prolonged postoperative pain control and remains one of the most frequently used neuraxial opioid agents in obstetric anesthesia [2,4].

Despite their established analgesic efficacy, the combined or sequential administration of neuraxial opioids through different routes may produce pharmacological interactions that remain incompletely characterized [5]. Contemporary reviews and obstetric anesthesia consensus guidance support the analgesic value of neuraxial opioids after cesarean delivery, while emphasizing dose-related adverse effects and structured postoperative monitoring [6,7]. In addition to well-recognized adverse effects such as pruritus, urinary retention, nausea, delayed respiratory depression, and hemodynamic changes, limited clinical and experimental observations have raised the possibility that opioids may influence uterine contractility [3,8,9].

Uterine atony is the leading cause of postpartum hemorrhage and one of the major causes of severe maternal morbidity worldwide [10,11]. Ineffective myometrial contraction prevents adequate hemostasis at the placental implantation site and can rapidly lead to life-threatening hemorrhage. Postpartum hemorrhage occurs across delivery modes, including cesarean delivery, and severe refractory atony requiring hysterectomy represents the extreme end of the hemorrhage spectrum. Although obstetric and surgical factors remain the primary contributors, experimental data suggest that opioids may interfere with myometrial activity through central and peripheral mechanisms, including modulation of oxytocin-mediated pathways [12,13].

In large epidemiological series, postpartum hemorrhage is reported in approximately 2–5% of deliveries, with cesarean delivery representing an important setting for severe hemorrhage because of higher baseline blood loss and surgical contributors [14]. Emergency peripartum hysterectomy for uncontrolled postpartum hemorrhage is uncommon, generally reported in the order of fewer than 1–5 per 1000 deliveries depending on population risk, cesarean rate, and abnormal placentation prevalence [15].

We report a case of severe refractory uterine atony after sequential epidural fentanyl and intrathecal morphine administration during labor analgesia and emergency cesarean delivery. The case is presented as a hypothesis-generating clinical observation. It highlights the difficulty of distinguishing anesthetic, obstetric, surgical, and hemorrhagic contributors in an individual patient and underscores the importance of rapid multidisciplinary management in severe postpartum hemorrhage.

## 2. Case Presentation

A 34-year-old primigravida (G1P0), with no significant past medical history, relevant comorbidities, or hereditary conditions, presented in February 2025 at 39 weeks and 4 days of gestation to the Obstetrics and Gynecology Unit of Pelican Hospital, Medicover, Romania. This case report was prepared in accordance with the Declaration of Helsinki (2024) and institutional requirements for publication of clinical case details. The Pelican Clinic Ethics Committee of Medicover Hospital approved publication under Approval No. 2421, dated 10 December 2019; the approval date refers to the institutional framework under which anonymized clinical case material is reviewed, while patient-specific consent for this 2025 case was obtained after the clinical event and before manuscript submission. Written informed consent for publication of the case details, accompanying clinical data, and intraoperative/pathological images was obtained from the patient.

She had been routinely followed throughout pregnancy, with no reported complications. The patient was of average build (weight: 68 kg, height: 1.64 m, body mass index: 25.3 kg/m^2^). She presented at 10:00 a.m. with painful but irregular uterine contractions that had begun less than one hour earlier. There was no associated vaginal bleeding, fluid loss, or sign of fetal distress.

The initial clinical examination revealed a hemodynamically stable patient with normal vital signs. Obstetric assessment indicated a singleton intrauterine pregnancy in cephalic presentation. The membranes were intact, and no spontaneous or induced amniotomy had occurred. Gynecological examination revealed a shortened, soft cervix that admitted the index finger, consistent with early labor. Uterine contractions exhibited an abnormal pattern and lacked coordinated sequential activity. Admission laboratory tests showed hemoglobin 12.7 g/dL, leukocyte count 7130/mm^3^, platelet count 131,000/mm^3^, and international normalized ratio 0.94. There were no clinical signs of infection or coagulopathy. Iron deficiency was noted without criteria for iron-deficiency anemia.

At 5:00 p.m., the cervix was dilated to 4 cm, with uterine contractions occurring at 5 min intervals and lasting 20–25 s. The patient described the contractions as significantly painful and requested epidural labor analgesia. An epidural catheter was placed successfully at 6:00 p.m., followed by an analgesic bolus of 8 mL containing 0.2% ropivacaine and fentanyl. The total fentanyl dose administered through the epidural route during labor was 100 mcg. Pain decreased to 2/10 on the numerical rating scale within 20 min, without motor blockade. Maternal hemodynamics remained stable, with blood pressure 128/75 mmHg and heart rate 92 beats/min. Continuous epidural analgesia was maintained with 0.2% ropivacaine at 7 mL/hour through an automated pump, and the epidural catheter remained in place for approximately 7 h in total.

By 8:00 p.m., reassessment revealed cervical dilation of 5 cm. Uterine contractions were hypotonic, occurring approximately every 5 min and lasting about 20 s. Labor augmentation was therefore initiated using intravenous oxytocin diluted as 10 IU in 500 mL isotonic saline and started at 8 drops/min using a 20 drops/mL drip factor, together with antispasmodic agents. Oxytocin augmentation was administered for 3 h and 30 min.

At 11:30 p.m., despite uterine stimulation, the cervix remained at 5 cm and had become hypertonic and non-compliant, consistent with spastic cervical dystocia. No spontaneous rupture of membranes or induced amniotomy occurred before the cesarean delivery decision. Because labor progression failed to improve after approximately 14 h from labor onset, emergency cesarean delivery was indicated.

At 0:05 a.m., spinal anesthesia was administered with 10 mg of 0.5% hyperbaric bupivacaine combined with 100 mcg of preservative-free intrathecal morphine. An adequate sensory block to the T6 dermatome and complete motor block were achieved. The patient developed mild post-spinal hypotension (92/65 mmHg), which responded to a 10 mg intravenous bolus of ephedrine.

The cesarean section began at approximately 0:10 a.m. and the neonate was delivered at approximately 0:12 a.m., seven minutes after administration of spinal anesthesia. The newborn weighed 3200 g, with Apgar scores of 9, 10, and 10 at 1, 5, and 10 min, respectively. The membranes had remained intact until delivery, and no abnormal amniotic fluid finding was documented. Placental separation and placental delivery occurred without difficulty; the placenta was delivered completely, and there was no intraoperative suspicion of retained placental tissue or abnormal placentation.

After delivery, uterine contraction was initially supported with oxytocin 20 IU. Severe uterine atony subsequently developed and was accompanied by significant hemorrhage. Escalated pharmacological management included ergometrine 0.2 mg, carbetocin 100 mcg, and misoprostol 800 mcg administered rectally. Continuous uterine massage was performed throughout this period. Conservative surgical measures included hemostatic suturing, including X-stitch placement, followed by B-Lynch uterine compression suture. Despite these interventions, adequate uterine tone and hemostasis were not achieved.

**Figure 1:**
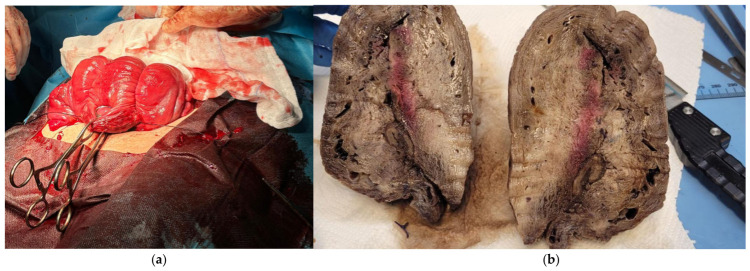
Sequential clinical illustrations of uterine atony management. (**a**) Intraoperative view of the exteriorized uterus. (**b**) Macroscopic pathological specimen of the uterus after hysterectomy. (**a**) Congestive, flaccid appearance of the uterine corpus despite previous conservative measures, with hemostatic clamps applied for temporary vascular control. (**b**) Thickened uterine walls with diffuse congestion and areas of internal hemorrhage.

Total blood loss was approximately 3800 mL. Blood loss was assessed using the suction canister and estimation from surgical swabs. Intraoperative blood product replacement included three units of packed red blood cells administered at 1:30 a.m., 2:25 a.m., and 3:15 a.m., and two units of fresh frozen plasma administered during the intraoperative period. Because hemorrhage persisted despite conservative pharmacological and mechanical measures, emergency total abdominal hysterectomy was indicated. The patient and her family were informed, and consent was obtained before proceeding. Figure 1a shows the intraoperative view of the exteriorized uterus during emergency hysterectomy performed for refractory uterine atony.

During preparation for hysterectomy, the patient became acutely hemodynamically unstable, requiring endotracheal intubation and conversion to general anesthesia at 2:15 a.m. Induction for intubation was performed with propofol 120 mg, fentanyl 0.15 mg, and rocuronium 35 mg. Anesthesia was maintained with sevoflurane at MAC 1.0–1.1. The total intraoperative general-anesthesia doses were fentanyl 0.35 mg and rocuronium 45 mg. Approximately 10 min after induction, the patient developed profound hypotension (60/35 mmHg). Norepinephrine was administered at a concentration of 80 mcg/mL, with an infusion rate between 200 and 700 mcg/hour, adjusted according to blood pressure; based on the patient’s 68 kg weight, this corresponded approximately to 0.05–0.17 mcg/kg/min.

The hysterectomy proceeded without further surgical complications. The surgical duration after intubation was approximately 90 min. Vasopressor support was gradually reduced toward the end of the procedure. The patient was extubated 20 min after cessation of anesthesia and transferred to the intensive care unit immediately postoperatively, once extubation had been completed and transfer was considered safe. ICU admission occurred at 4:15 a.m.

In the ICU, norepinephrine was discontinued after 1 h and 20 min. The patient was alert, oriented, and hemodynamically and respiratorily stable, with no neurological deficit. Postoperative correction of anemia and coagulopathy included one unit of cryoprecipitate at 5:00 a.m., one unit of fresh frozen plasma at 5:35 a.m., two additional units of packed red blood cells at 5:10 a.m. and 7:15 a.m., and four platelet concentrate units at 8:35 a.m.

Her recovery was uneventful, and she was transferred to the obstetric ward in stable condition. At hospital discharge on the eighth postoperative day, hemoglobin was 9.2 g/dL, platelet count was 261,000/mm^3^, and leukocyte count was 7630/mm^3^. The patient was counseled on the clinical events and outcome, including the implications of hysterectomy for future fertility. Psychological support and follow-up were arranged as part of comprehensive postnatal care. Figure 1b presents the macroscopic appearance of the uterus after hysterectomy, showing diffuse hemorrhagic changes and loss of myometrial tone.

A comprehensive chronological summary of the clinical course, anesthetic exposure, hemorrhage management, and postoperative outcome is provided in Table 1. Uterotonic/hemostatic measures and transfusion support are summarized separately in Table 2 and Table 3.

## 3. Discussion

Postpartum hemorrhage is a life-threatening obstetric emergency that occurs across delivery modes, including cesarean delivery. Uterine atony accounts for most postpartum hemorrhage cases and remains a major cause of maternal morbidity and mortality worldwide [14,15]. Established contributing factors include prolonged or dysfunctional labor, uterine overdistension, chorioamnionitis, multiparity, prior uterine surgery, placental abruption, retained placental tissue, uterine-relaxing medications, cesarean delivery, and anesthetic exposure. In the present case, the clinically relevant factors included dysfunctional labor with cervical dystocia, oxytocin augmentation, emergency cesarean delivery, sequential neuraxial opioid exposure, subsequent exposure to general anesthetic agents, severe hemorrhage, and evolving coagulopathy.

**Figure 2:**
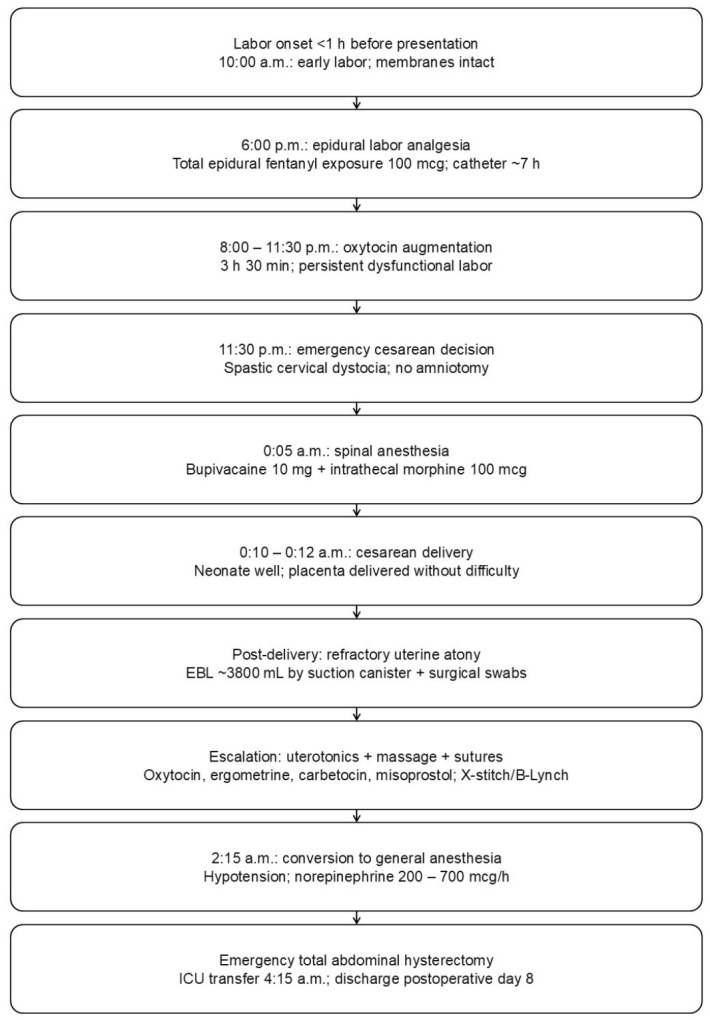
Revised flowchart of the sequential clinical management of the reported case, including labor progression, anesthetic exposure, hemorrhage escalation, transfusion support, hysterectomy, and postoperative outcome.

The clinical decision-making process and escalation of interventions are visually summarized in Figure 2. The patient had no history of uterine surgery, multiparity, hypertensive disease, infection, suspected placental abruption, retained placenta, or abnormal placentation. Placental separation was uncomplicated. These observations reduce the likelihood of several common etiologies but do not eliminate the possibility that multiple perioperative factors acted together.

This case does not prove that neuraxial opioids caused uterine atony. It does, however, raise a biologically plausible question because severe uterine atony occurred after epidural fentanyl exposure during labor and intrathecal morphine administration for cesarean anesthesia. Intrathecal morphine is commonly used for postoperative analgesia in cesarean delivery because of its long duration of action and pharmacokinetic properties [16]. Its potential effect on uterine tone in vivo remains insufficiently defined.

Experimental studies suggest that μ-opioid receptors are present in human myometrium and that opioids may influence contractility of isolated pregnant uterine muscle [17]. Fentanyl and morphine have shown dose-dependent inhibitory effects in in vitro myometrial preparations [17,18]. Kayacan et al. reported reduced myometrial contractility after opioid exposure, supporting a potential local pharmacodynamic effect in addition to central analgesic effects [18]. These experimental observations provide mechanistic plausibility but cannot be directly extrapolated to prove causation in a single clinical case.

In the present case, the patient received epidural fentanyl before intrathecal morphine. The total epidural fentanyl exposure was 100 mcg, and intrathecal morphine was administered shortly before delivery. A cumulative or sensitizing effect on uterine contractility is possible but remains speculative. Epidural fentanyl is highly lipophilic, and although systemic absorption is generally limited, some maternal systemic exposure may occur [19]. Morphine may also influence oxytocin release or oxytocin-mediated uterine activity in experimental settings [20]. These observations are relevant because the uterus responded poorly to uterotonic therapy, but they should be interpreted cautiously.

Opioid-related interference with oxytocin signaling has been proposed in experimental literature. Oxytocin-mediated uterine contraction depends on oxytocin receptor activation and downstream intracellular calcium mobilization pathways [21]. In vitro and animal studies suggest that morphine may weaken oxytocin-stimulated uterine activity [20]. In this case, the reduced response to oxytocin and other uterotonics could be compatible with such a mechanism, but alternative explanations, including dysfunctional labor, cesarean delivery, hemorrhagic shock, and evolving coagulopathy, remain plausible.

The main strength of this report is the detailed chronological description of labor analgesia, anesthetic exposure, hemorrhage progression, uterotonic escalation, transfusion therapy, and surgical management. The case may help clinicians recognize that refractory atony can progress rapidly even after apparently uncomplicated placental separation and that early escalation is essential when standard uterotonic therapy fails.

This report has several limitations. First, as a single case report, it cannot establish causality between sequential neuraxial opioid administration and refractory uterine atony. Second, several non-opioid contributors were present, including dysfunctional labor, oxytocin augmentation, emergency cesarean delivery, severe hemorrhage, evolving coagulopathy, and subsequent exposure to general anesthetic agents. Third, uterine tone was assessed clinically rather than by objective measurement of myometrial contractility. Fourth, maternal opioid concentrations, oxytocin receptor assessment, and myometrial receptor analysis were not available. Fifth, blood loss was measured using suction canister volume and estimated from surgical swabs, which may be less precise than fully quantitative gravimetric methods. Therefore, the temporal association between sequential neuraxial opioid administration and uterine atony should be considered hypothesis-generating rather than confirmatory.

In clinical practice, anesthesiologists and obstetricians should work collaboratively to identify and manage uterine atony promptly. Early recognition, rapid use of multimodal uterotonics, continuous uterine massage, timely mechanical and surgical measures, activation of transfusion support, and awareness of potentially contributing anesthetic factors are important steps in reducing severe postpartum hemorrhage-related morbidity.

## 4. Conclusions

This case highlights the complexity of diagnosing and managing refractory uterine atony after emergency cesarean delivery. Sequential epidural fentanyl and intrathecal morphine exposure occurred before the onset of severe atony, and a contributory role cannot be excluded; however, causality cannot be inferred because multiple obstetric, surgical, anesthetic, and hemorrhagic factors were present. Clinicians should remain vigilant when uterotonic response is suboptimal, rapidly escalate pharmacological and mechanical interventions, activate transfusion support early, and involve a multidisciplinary team. Further clinical and experimental studies are required to clarify whether neuraxial opioids influence myometrial contractility in obstetric patients.

## Figures and Tables

**Table 1:** Chronological summary of labor, anesthetic exposure, hemorrhage management, and postoperative course.

Time/Interval	Obstetric Status	Anesthetic/Drug Exposure	Outcome/Comment
<1 h before 10:00 a.m.; presentation at 10:00 a.m.	Painful but irregular contractions; no bleeding, no fluid loss, no fetal distress.	No analgesia/anesthesia at presentation.	Early labor; membranes intact.
5:00 p.m.	Cervix 4 cm; contractions every 5 min, lasting 20–25 s.	Epidural analgesia requested.	Pain significant; maternal hemodynamics stable.
6:00 p.m.–approximately 1:00 a.m.	Labor analgesia period.	Epidural catheter in place approximately 7 h; total epidural fentanyl exposure 100 mcg; 0.2% ropivacaine infusion at 7 mL/h.	Pain decreased to 2/10; no motor block.
8:00–11:30 p.m.	Cervix 5 cm; hypotonic contractions.	Oxytocin infusion for 3 h 30 min, diluted 10 IU in 500 mL isotonic saline, started at 8 drops/min.	Labor augmentation attempted.
11:30 p.m.	Cervix remained 5 cm, hypertonic and non-compliant; membranes still intact.	No spontaneous or induced amniotomy before cesarean decision.	Emergency cesarean delivery indicated for failed labor progression/spastic cervical dystocia after approximately 14 h from labor onset.
0:05 a.m.	Preparation for emergency cesarean delivery.	Spinal anesthesia: 10 mg 0.5% hyperbaric bupivacaine + 100 mcg preservative-free morphine.	T6 sensory block; mild hypotension treated with ephedrine 10 mg IV.
0:10–0:12 a.m.	Cesarean section started; neonate delivered approximately 7 min after spinal anesthesia.	Neonatal outcome: 3200 g; Apgar 9, 10, 10.	Membranes intact until delivery; no abnormal amniotic fluid finding documented; placenta delivered without difficulty.
Post-delivery	Severe uterine atony and hemorrhage.	Oxytocin, ergometrine, carbetocin, misoprostol, continuous uterine massage, X-stitch, and B-Lynch suture.	Conservative pharmacological and mechanical measures failed.
1:30–3:15 a.m.	Ongoing hemorrhage; total blood loss approximately 3800 mL.	PRBCs at 1:30, 2:25, and 3:15 a.m.; FFP during intraoperative period.	Blood loss measured using suction canister and estimated from surgical swabs.
2:15 a.m.	Hemodynamic instability during preparation for hysterectomy.	General anesthesia: propofol 120 mg, fentanyl 0.15 mg, rocuronium 35 mg; sevoflurane MAC 1.0–1.1.	Profound hypotension required norepinephrine 80 mcg/mL at 200–700 mcg/h.
Approximately 3:45–4:15 a.m.	Emergency total abdominal hysterectomy completed; patient extubated.	Total general anesthesia as above	Transferred to ICU at 4:15 a.m. after safe extubation.
ICU period	Postoperative monitoring and correction of anemia/coagulopathy.	Cryoprecipitate, FFP, PRBCs, and platelets administered.	Norepinephrine stopped after 1 h 20 min; stable clinical course.
Postoperative day 8	Clinical recovery.	No further anesthesia-related intervention.	Discharged in stable condition; counseling and psychological follow-up arranged.

FFP, fresh frozen plasma; ICU, intensive care unit; IV, intravenous; MAC, minimum alveolar concentration; PRBCs, packed red blood cells.

**Table 2:** Uterotonic and hemostatic interventions for refractory uterine atony.

Order/Time	Intervention	Dose/Route	Response/Comment
Immediately after delivery	Oxytocin	20 IU IV	Initial uterine contraction support; atony subsequently developed.
Escalation after atony recognized	Ergometrine	0.2 mg IV	Insufficient response.
Escalation after persistent atony	Carbetocin	100 mcg IV	Insufficient response.
Escalation after persistent atony	Misoprostol	800 mcg rectally	Insufficient response.
Throughout hemorrhage management	Continuous uterine massage	Manual intervention	Performed continuously during pharmacological escalation.
Conservative surgical step	Hemostatic suturing, including X-stitch	Surgical intervention	Hemostasis remained inadequate.
Conservative surgical step	B-Lynch uterine compression suture	Surgical intervention	Failed to achieve durable hemorrhage control.
Definitive surgical management	Emergency total abdominal hysterectomy	Surgical intervention	Performed after failure of conservative pharmacological and mechanical measures.

IU, international units.

**Table 3:** Transfusion and coagulation support.

Time/Period	Blood Product	Units/Dose	Phase/Comment
1:30 a.m.	Packed red blood cells	1 unit	Intraoperative transfusion.
2:25 a.m.	Packed red blood cells	1 unit	Intraoperative transfusion.
3:15 a.m.	Packed red blood cells	1 unit	Intraoperative transfusion.
Intraoperative period	Fresh frozen plasma	2 units	Administered during ongoing hemorrhage; exact individual unit times not recorded.
5:00 a.m.	Cryoprecipitate	1 unit	Postoperative ICU correction of coagulopathy.
5:10 a.m.	Packed red blood cells	1 unit	Postoperative ICU transfusion.
5:35 a.m.	Fresh frozen plasma	1 unit	Postoperative ICU correction of coagulopathy.
7:15 a.m.	Packed red blood cells	1 unit	Postoperative ICU transfusion.
8:35 a.m.	Platelet concentrates	4 units	Postoperative ICU correction of thrombocytopenia/coagulopathy.

FFP, fresh frozen plasma; ICU, intensive care unit.

## Data Availability

The relevant clinical information is included in this article. Additional de-identified details are not publicly available because of patient privacy restrictions but may be made available by the corresponding author upon reasonable request and in accordance with institutional requirements.
